# MEG Responses to the Perception of Global Structure within Glass Patterns

**DOI:** 10.1371/journal.pone.0013865

**Published:** 2010-11-05

**Authors:** Jennifer B. Swettenham, Stephen J. Anderson, Ngoc J. Thai

**Affiliations:** 1 CUBRIC, School of Psychology, Cardiff University, Cardiff, United Kingdom; 2 The Wellcome Trust Laboratory for MEG Studies, School of Life and Health Sciences, Aston University, Birmingham, United Kingdom; Cuban Neuroscience Center, Cuba

## Abstract

**Background:**

The perception of global form requires integration of local visual cues across space and is the foundation for object recognition. Here we used magnetoencephalography (MEG) to study the location and time course of neuronal activity associated with the perception of global structure from local image features. To minimize neuronal activity to low-level stimulus properties, such as luminance and contrast, the local image features were held constant during all phases of the MEG recording. This allowed us to assess the relative importance of striate (V1) versus extrastriate cortex in global form perception.

**Methodology/Principal Findings:**

Stimuli were horizontal, rotational and radial Glass patterns. Glass patterns without coherent structure were viewed during the baseline period to ensure neuronal responses reflected perception of structure and not changes in local image features. The spatial distribution of task-related changes in source power was mapped using Synthetic Aperture Magnetometry (SAM), and the time course of activity within areas of maximal power change was determined by calculating time-frequency plots using a Hilbert transform. For six out of eight observers, passive viewing of global structure was associated with a reduction in 10–20 Hz cortical oscillatory power within extrastriate occipital cortex. The location of greatest power change was the same for each pattern type, being close to or within visual area V3a. No peaks of activity were observed in area V1. Time-frequency analyses indicated that neural activity was least for horizontal patterns.

**Conclusions:**

We conclude: (i) visual area V3a is involved in the analysis of global form; (ii) the neural signature for perception of structure, as assessed using MEG, is a reduction in 10–20 Hz oscillatory power; (iii) different neural processes may underlie the perception of horizontal as opposed to radial or rotational structure; and (iv) area V1 is not strongly activated by global form in Glass patterns.

## Introduction

To perceive the world around us, our visual system must construct global form from the many local visual cues present across space. Perception of global form from simple features, such as edges and lines, is then the foundation for object recognition. While the first psychophysical and computational models of visual integration date back to the pioneering work of Gestalt psychologists, it is only recently – with the advance of neuroimaging tools – that the neural signature associated with global form perception has been investigated in humans.

Neuroimaging studies have indicated a number of discrete brain regions where global form processing may occur. Using fMRI, activation within ventral (putative area V4) and dorsal (putative area V3) cortical areas has been reported in response to global structure within Glass patterns [1], concentrically oriented lines [2], and concentrically oriented Gabor arrays [3]. The involvement of area V4 in intermediate form processing is also supported by compromised pattern discrimination in a patient with a brain lesion in this area [4]. Another candidate area for global form processing is V3a, so named not because of its similarity to area V3 but because it lies between the previously identified areas V3 and V4 [5], [6]. Neurons in V3a have significantly larger receptive fields compared with neurons in earlier visual areas [7], making them ideally suited to integrate signal over space. Recent fMRI studies support the involvement of V3a in form processing, with evidence that it is differentially activated by concentric versus parallel line patterns [8] and by spiral Glass patterns [9]. Activation of an area slightly inferior to V3a has also been reported in an fMRI experiment using static Glass pattern stimuli [1]. Unlike its macaque counterpart, human V3a is also known to be relatively motion-selective [7], and both V3a and V4 are sensitive to motion edges [10].

There is evidence that the neural processes underlying global form perception may be pattern specific. For example, using fMRI, Wilkinson et al. [11] reported that human V4 shows a larger metabolic (BOLD) response to rotational and radial gratings than to linear gratings. This is supported by electroencephalographic studies showing larger neural responses to rotational and radial patterns than to translational (linear) patterns [12], [13]. These results are in general agreement with psychophysical reports of higher detection thresholds for translational structure than radial or rotational structure [14]–[19]. To account for such findings, it has been postulated that translational patterns may be processed locally while more complex patterns are processed globally [18], .

To investigate the neural response to global structure from local image features, and address whether this response varies between pattern types, we used the neuroimaging technique of MEG in combination with radial, rotational and horizontal Glass pattern stimuli [22]. MEG provides a direct measure of neural function in that the recorded signals reflect electrical activity of cortical pyramidal cells [see 23]. As with electrical measures of brain function (electroencephalography, EEG), magnetic measures can be recorded on a time scale that is compatible with brain physiology (i.e. milliseconds). This is a significant advantage over slower metabolic measures of brain function (e.g. PET, fMRI). Unlike electrical currents, magnetic fields are little distorted by brain tissue or bone and thus MEG can be used to determine sites of neural activity with greater resolution than EEG. However, MEG does not produce an anatomical image of the brain and so for source localization an anatomical MR scan for each individual is required for co-registration with the MEG data. A consideration with the beamformer analysis technique we use here (see below) is that it produces an inhomogeneous spatial resolution across the brain, with resolution being directly related to the amplitude of the activity [24]. Consequently, group analysis, in which individuals' data are spatially normalized in an attempt to overlay brain regions, may fail to detect large peaks of activity which have high spatial resolution and do not overlap simply due to normal levels of individual anatomical variability. Alternatively, group analysis may reveal regions of relatively low activity which overlap due to their low spatial resolution. Differences in results between individual and group level analysis have been reported [25], but fortunately for MEG studies the high signal-to-noise ratios often achieved allow meaningful analysis at the individual level.

This experiment was designed to find the neural response to global form, and to minimise as much as possible any other factors that might produce a response. To achieve this, observers viewed dynamic Glass patterns during both the active and passive phases of the experiment. Such stimuli, in which patterns are replotted periodically, allow structure type and coherence to be changed between frames without affecting local cues such as dot density or mean luminance. In our experiments, the Glass patterns were replotted every 10 ms to keep flicker constant throughout the trials. In addition, the onset and offset of coherent form was ramped to eliminate abrupt visual changes between structure and no-structure.

The various neural models of global form perception all posit the need for multiple stages of processing across different regions of the brain. The process by which disparate neural areas interact is not known, though it has been postulated that functional integration within and between areas could be accomplished via changes (either increase or decrease) in synchronous cortical activity [for reviews see 26]–[28]. To further assess the functional role of synchronous activity in global form perception, we analyzed the MEG data using the beamforming technique of Synthetic Aperture Magnetometry (SAM), which is ideally suited for the analysis of event-related changes in cortical rhythms [29]–[38].

## Methods

### Ethics Statement

This study adhered to the tenets of the Declaration of Helsinki and was approved by the Ethical Committee of Aston University. All observers gave written informed consent.

### Participants

A total of eight observers (four male and four female, aged 25–48 years) consented to participate in the study. All observers had normal or corrected-to-normal vision and no history of neurological dysfunction or injury. All observers had previously acquired anatomical MR scans.

### Stimuli

**Figure 1:**
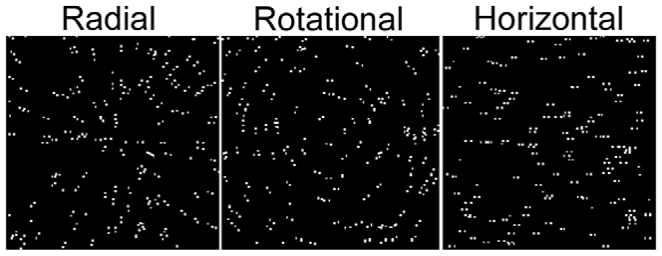
Schematic illustration of Glass patterns.

Glass patterns with radial, rotational or horizontal structure were created by randomly placing 150 white dots (70.2 cd.m^−2^, 0.04×0.04 deg) on a black background (5.0×10^−3^ cd.m^−2^) within a square window (8.25×8.25 deg), and then providing each dot with a partner whose relative position was determined by a common rule (Figure 1). The proportion of dot pairs oriented to the common rule defined the coherence level. Remaining dot pairs were plotted at randomly selected orientations. To create radial patterns, the partner dot was positioned on the radial (i.e. aligned with the original dot and the centre of the field) and further from the centre than the original dot. Rotational patterns were created by placing the partner dot orthogonal to the radial and clockwise from the original dot. Horizontal patterns were created by placing the partner dot to the right of the original dot. The centre-to-centre spacing within each dot pair was 0.18 deg. Dot density remained constant across the stimulus. All dot arrays were re-plotted every 10 ms, giving each pattern a dynamic appearance.

**Figure 2:**
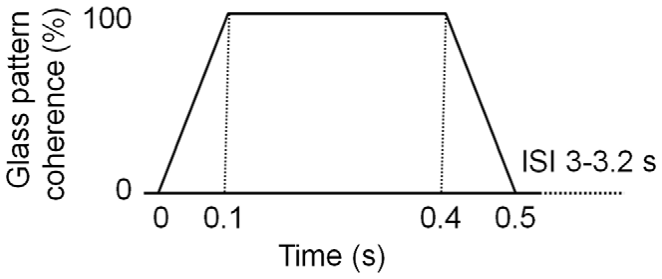
Schematic illustration of coherence changes within the Glass pattern during a trial.

Each trial was between 3.5 and 3.7 s and had an initial period of Glass pattern with radial, rotational or horizontal structure, followed by an inter-stimulus interval (ISI) of Glass pattern without structure. The next trial followed immediately such that the screen was never blank, with the last 1 s of the ISI forming the baseline for the next coherent phase. Figure 2 shows schematically the experiment design: on each trial, the coherence of the Glass pattern increased linearly from zero to 100% over 100 ms, remained at 100% coherence for 300 ms, then decreased linearly to zero over 100 ms. The ISI varied between 3000 and 3200 ms, during which time the Glass pattern had zero coherence. Dot density and mean luminance remained constant throughout the entire display period. During recording, a red, central fixation target was continuously visible in the centre of the screen.

### Recording procedure

#### Central viewing

MEG data were recorded in a dimly-lit magnetically shielded room using a 151-channel whole-head CTF imaging system (VSM MedTech Ltd., Coquitlam, Canada) in a single non-averaged run at a sampling rate of 625 Hz, during which 50 trials of each pattern type were presented in pseudo-random order. Collection of each dataset took approximately nine minutes. The display monitor was outside the shielded room and viewed through a hole in the room's wall via a front-surfaced mirror within the room. The linearised Sony GDM F520 monitor was controlled using a VSG2/5 graphics card from Cambridge Research Systems (Rochester, UK). The screen size was 1024 by 768 pixels and the frame rate was 100 Hz. The screen was viewed monocularly and the non-viewing eye was occluded with a dark patch. The optical viewing distance was 2 m. Observers were fitted with three electromagnetic head coils, which were localised relative to the MEG system before and after each recording session. Following recording, a three dimensional digitiser (Polhemus Isotrak) was used to determine the position of these coils relative to the surface of each observer's head, and this head surface was matched to their own MRI-defined head shape using the software Align (www.ece.drexel.edu/ICVC/Align/align11.html).

Offline, each data set was band-pass-filtered using a fourth-order bi-directional IIR Butterworth filter into 10 Hz width frequency bands between zero and 100 Hz. Evenly spaced frequency bands were used so that the accuracy of covariance matrix estimation would be equal for each frequency band [30]. The SAM (synthetic aperture magnetometry) beamformer algorithm was used to create differential images of source power (pseudo-*T* statistics) for 1 s of baseline (−1 to 0 s) compared with 1 s of visual stimulation (0 to 1 s). Time windows for baseline estimation were of equal duration to the time window of interest to achieve balanced covariance estimation. Pilot analyses using different time windows showed 1 s to be optimal for maximising power responses. Details of the calculation of SAM pseudo-*T* source image statistics are described in detail in a number of sources [31]–[35]. For source localization, a multiple, local-spheres-forward model was derived by fitting spheres to the brain surface extracted by BET [39]. Estimates of the three-dimensional distribution of source power were derived for each observer's whole head at 3 mm isotropic voxel resolution. SAM images were visualized using mri3dX (https://cubric.psych.cf.ac.uk/Documentation/mri3dX/).

For spatial locations of interest, activation time courses were calculated as if a sensor or an electrode were at that position, i.e. a virtual electrode. Time courses were constructed using SAM beamformer coefficients obtained using the individual condition covariance matrices band-pass filtered between zero and 100 Hz [34]. For each of these virtual-electrode time courses, time-frequency spectrograms were generated by determination of the time-varying amplitudes at each sample frequency. These envelopes were formed from the amplitude of the analytic signal derived using the Hilbert Transform. The resulting spectrograms were calculated separately for each trial and then averaged in order to reveal both induced and evoked responses. Here, we present spectrograms as a percentage change from the mean baseline power at each frequency.

#### Eccentric viewing

For two observers (male, aged 47–48 years), the first experiment was repeated using eccentric viewing. This controlled for the possibility that the detectability of neural activity in early visual areas to centrally-viewed stimuli was compromised by self-cancelling magnetic fields (generated by neurons on geometrically opposing banks of the calcarine and inter-hemispheric midline). In this experiment, the Glass patterns were confined to the lower, left quadrant of the visual field, and comprised 38 dot pairs covering an area of 4.12 deg^2^. The edges of the pattern were 0.5 deg from the horizontal and vertical meridians. Other details were as reported above.

## Results

### Central viewing of Glass patterns

**Figure 3:**
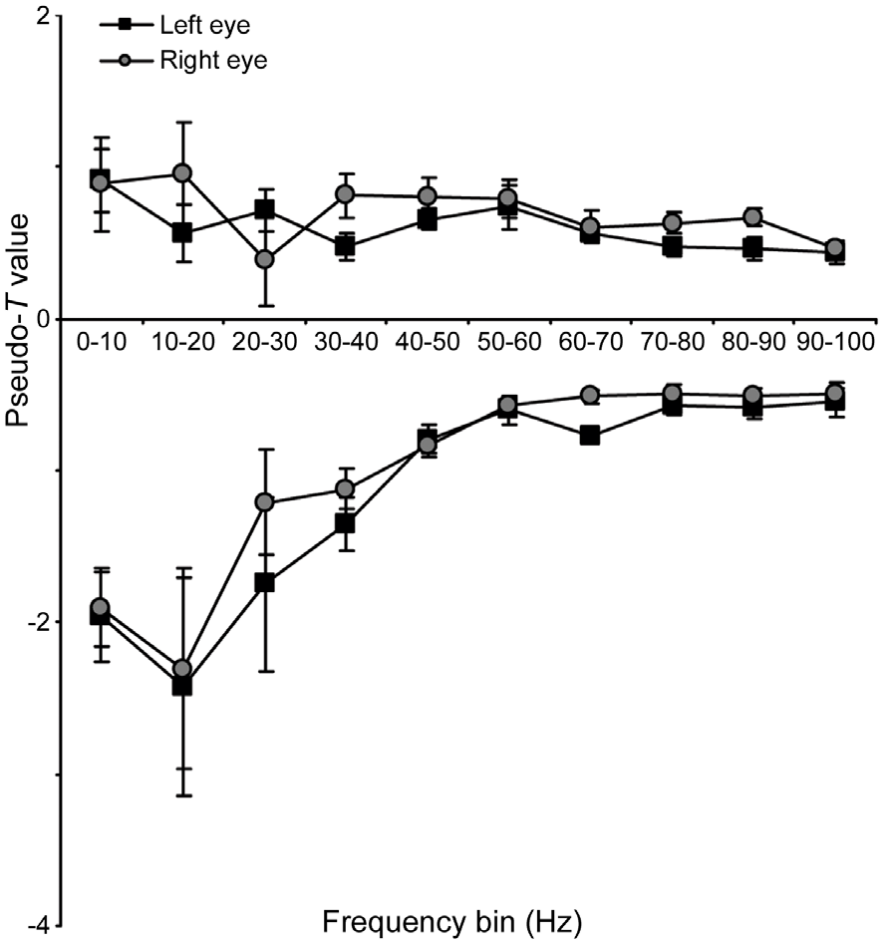
Largest energy changes in each 10 Hz frequency bin of SAM analysis. For each of the SAM analyses, performed in 10 Hz frequency bins for 1 s pre- vs. 1 s post-onset of coherent structure in the Glass pattern, the mean (n = 8) of the largest negative and positive pseudo-*T* value observed in occipital cortex is plotted. Negative and positive pseudo-*T* values represent decreases and increases respectively in energy compared with baseline (viewing Glass pattern with random structure). Error bars represent one standard error of the mean.

SAM analyses were conducted in 10 Hz bins from zero to 100 Hz. Figure 3 shows how the magnitude of the largest power changes in occipital cortex varies with frequency. For each observer (n = 8) the largest change (increase or decrease) in source power (pseudo-*T* statistic) within the occipital cortex was found for each frequency band and for each eye viewing. Each point is the mean (n = 8) of these maximal values: positive and negative pseudo-*T* values represent increases and decreases in energy, respectively. Figure 3 shows there was no difference between the largest changes in source power for left- versus right-eye viewing, and that the predominant change in response to the perception of Glass patterns was event-related power decreases within the 10–20 Hz range. The spatial location and time-course of cortical activity within this frequency band are explored further below.

**Figure 4:**
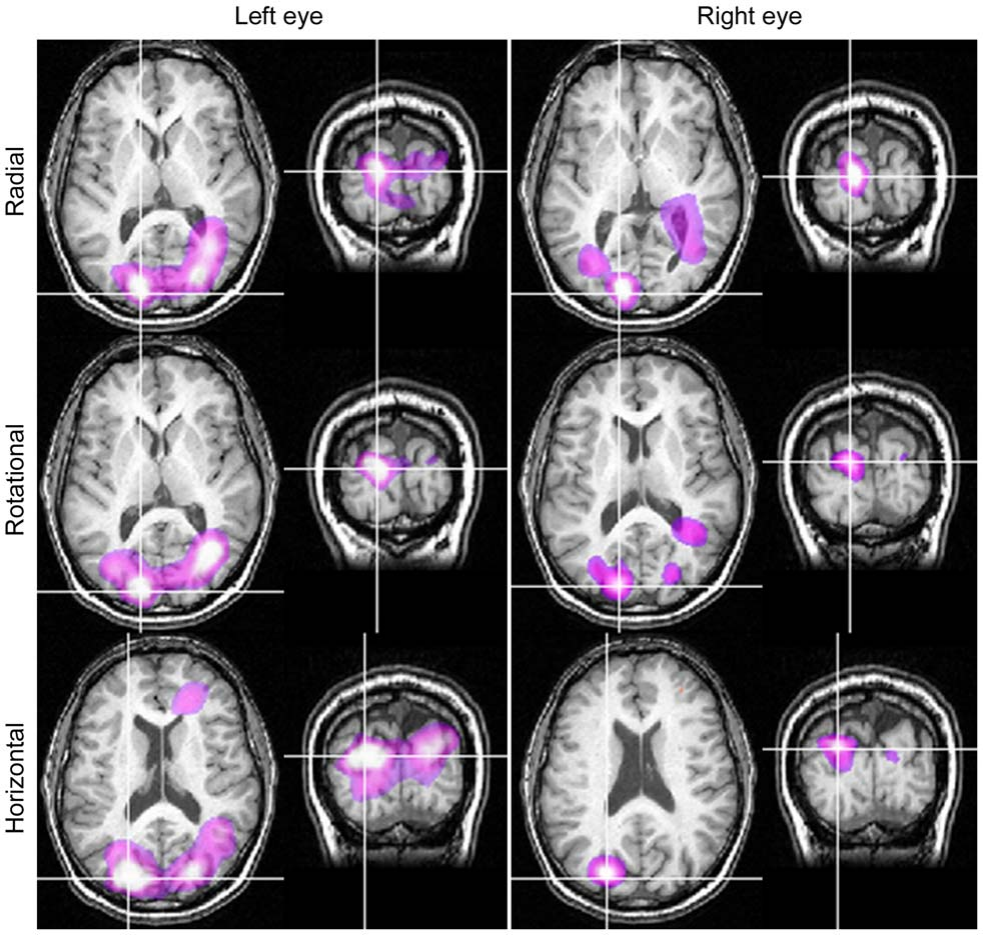
SAM images (n = 1) showing similar regions of activity irrespective of viewing eye or pattern type. SAM images for Observer A showing statistical estimates of power changes within the 10–20 Hz frequency band with 1 s time windows. The colour indicates the amplitude of the pseudo-*T* statistic (2<*T*<6) with blue/purple colours representing power decreases. SAM images are overlaid on the individual's structural MR, with axial and coronal slices through the voxel with the largest power change.

**Figure 5:**
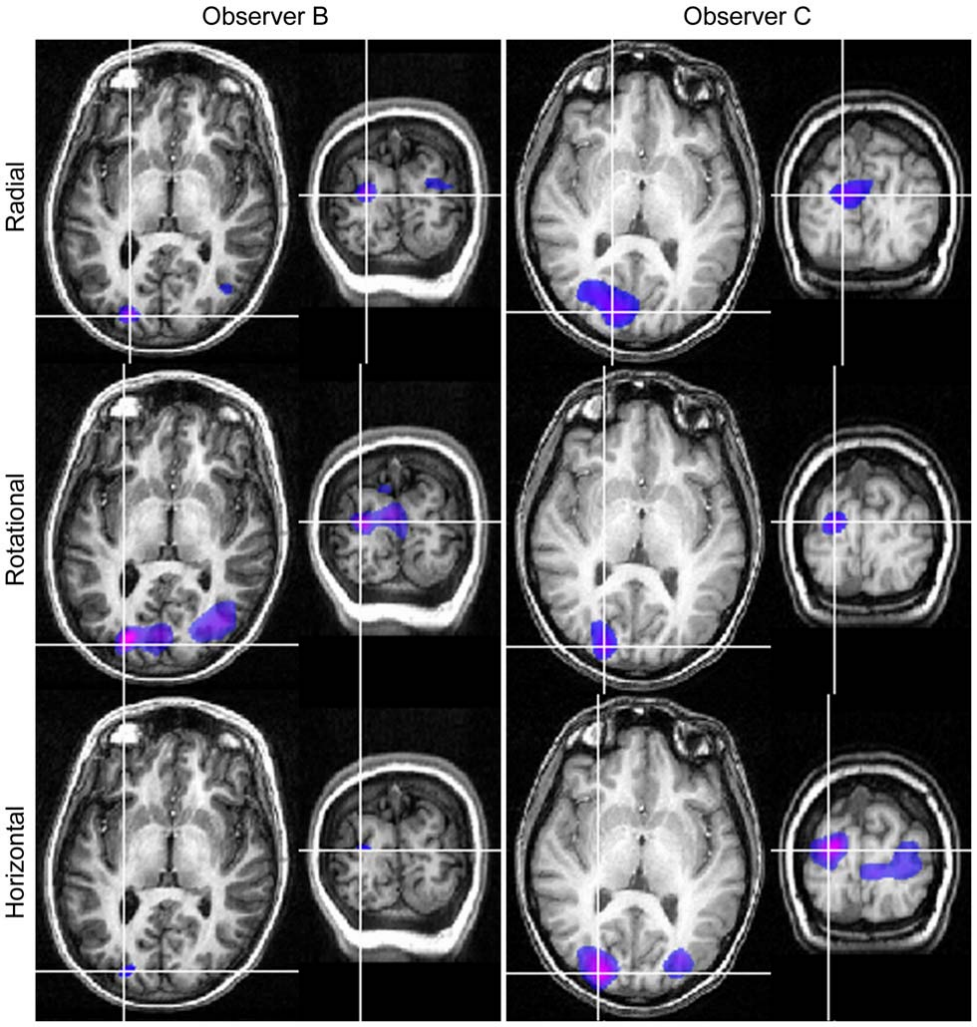
SAM images for two observers showing similar regions of activity irrespective of pattern type. SAM images for Observers B (left column) and C (right column), left eye viewing, showing statistical estimates of power changes within the 10–20 Hz frequency band with 1 s time windows. The colour indicates the amplitude of the pseudo-T statistic (2<*T*<6) with blue/purple colours representing power decreases. SAM images are overlaid on each individual's structural MR, with axial and coronal slices through the voxel with the largest power change.

Within the 10–20 Hz band, SAM analyses revealed regions of activity with pseudo-*T* values greater than two within extrastriate cortex in six (out of eight) participants. Four observers showed activations when viewing with each eye, and one observer only with right-eye viewing and one only with the left-eye viewing. All remaining analyses are based on these six participants. Figure 4 shows, for one such participant, SAM images of 10–20 Hz power changes for the passive perception of radial, rotational and horizontal Glass patterns. For each pattern type, results are shown on axial and coronal MRI slices for both right- and left-eye viewing: the white-purple colours indicate a relative decrease in oscillatory power. The regions of power change are similar for each eye. For each stimulus type, the voxel of maximal power change (the peak voxel), demarcated by cross-hairs in each panel, corresponds most closely to area V3a [40]. Similarly, Figure 5 shows, for left-eye viewing only, SAM images of 10–20 Hz power changes for two further participants. Again, the peak voxel was near area V3a for each observer. Table 1 shows the location of principal activity for the six participants who showed activations.

**Table 1:** Locations of principal peaks in activity.

Observer	Eye	Pseudo *T*	x	Y	z
A	Left	*−7.4	−12.9	−88.2	15.3
		−6.3	25.7	−80.1	17.5
	Right	*−4.9	−12.9	−88.2	15.3
B	Left	*−4.1	23.3	−85.3	18.1
	Right	−3.5	25.0	−77.9	20.5
		*−3.3	−19.6	−82.2	13.3
C	Left	*−3.7	−13.8	−82.2	22.2
		−2.2	24.8	−87.0	4.1
	Right	*−3.1	−18.0	−82.5	22.5
D	Left	*−2.2	−18.4	−86.7	4.1
	Right	*−2.0	−18.5	−87.6	−0.7
E	Left	*−2.3	−15.8	−83.2	9.0
F	Right	*−4.5	22.6	−84.5	−9.3

Pseudo-*T* values and co-ordinates for peaks of activity in response to viewing coherent form in Glass patterns. Each observer's individual MRI was scaled to MNI space using mri3dX (https://cubric.psych.cf.ac.uk/Documentation/mri3dX/).

**Figure 6:**
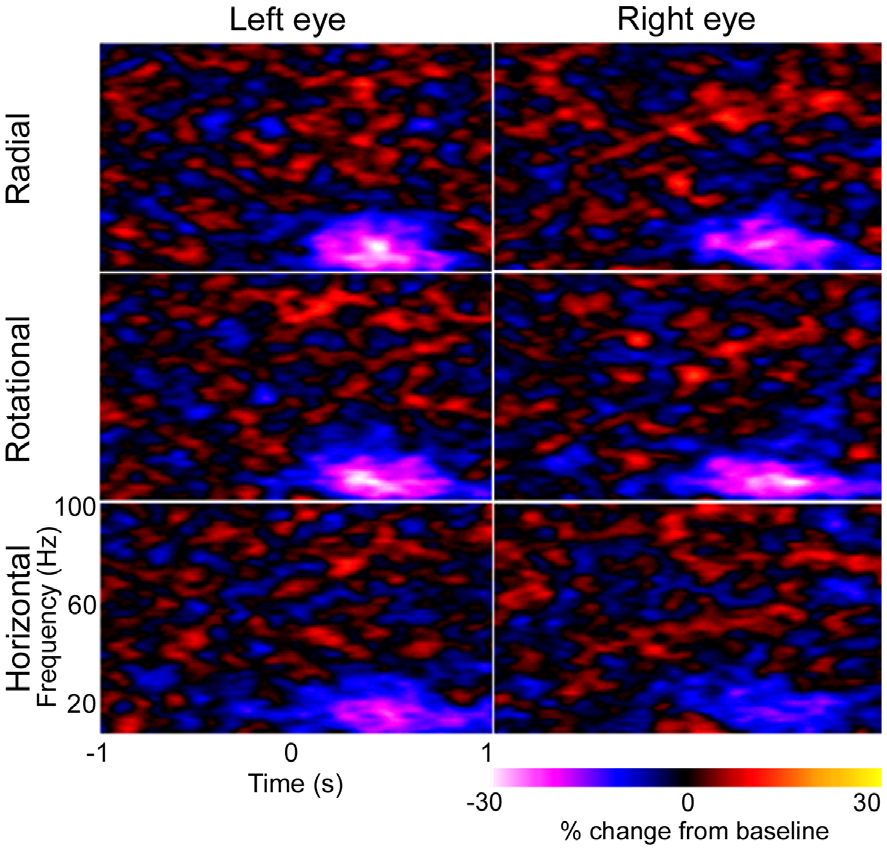
Mean (n = 5) time-frequency plots. Mean (n = 5) time-frequency plots are shown for virtual electrodes placed at the location of maximal power change as determined using SAM (10–20 Hz, 1 s time windows). All virtual electrodes were in visual cortex.

*Locations used in time-frequency analysis (Figs 5 and 6).

**Figure 7:**
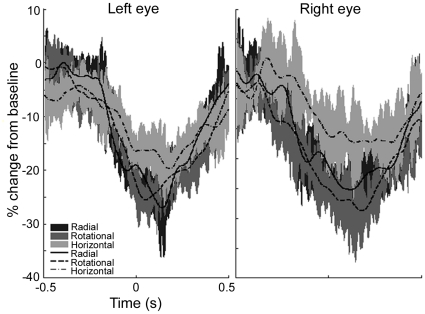
Temporal evolution of the 10–20 Hz group average (n = 5) response. Group average (n = 5) responses showing the temporal evolution of the 10–20 Hz cortical response in visual cortex to viewing coherent structure. The shaded regions indicate the standard error of the mean across participants.

The time and frequency components of cortical activity associated with the perception of Glass patterns were calculated for each participant. Figure 6 shows the mean (n = 5) time–frequency plots for both left- and right-eye viewing of each stimulus type, as determined using virtual electrodes at the location of maximal power change (from Figures 4–5, Table 1). For each plot the Glass pattern increased in coherence from time zero and returned to random coherence at 500 ms. Purple-white colours represent decreases in energy compared with the mean baseline power at each frequency. These plots illustrate a decrease in 10–20 Hz energy that is maintained for most of the first second following coherence onset, i.e. the time-frequency plots do not show a response that varies greatly (in the time or frequency domain) from the SAM analyses (10–20 Hz band and 1 s time windows) used to select the voxels of interest. The plots do not reveal any other time-frequency component of interest. A decrease in 10–20 Hz energy was observed for all pattern types but the magnitude of the reduction to horizontal patterns appeared less marked than for either radial or rotational patterns. This is also shown in Figure 7, where the temporal evolution of 10–20 Hz activity at the peak locations is shown for each pattern type. For both right- and left-eye viewing, there is a trend for the energy change to be less for horizontal patterns than for either radial or rotational patterns.

### Eccentric viewing of Glass patterns

**Figure 8:**
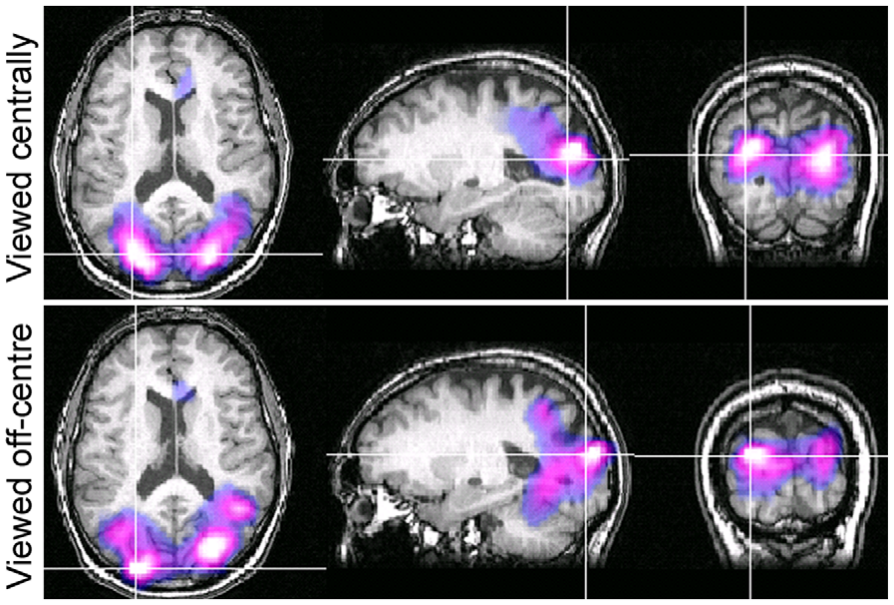
SAM images showing similar regions of activation for central and off-centre viewing. SAM images for Observer A showing statistical estimates of power changes, from the summed responses to rotational, radial and horizontal Glass patterns, within the 10–20 Hz frequency band with 1 s time windows. The top panels show responses when Glass patterns were centrally viewed and the lower panels show responses when Glass patterns were viewed in the lower-left visual field. The colour indicates the amplitude of the pseudo-*T* statistic (2<*T*<6) with blue/purple colours representing power decreases. SAM images are overlaid on the individual's structural MR, with slices through the voxel with the largest power change.

Glass patterns were viewed eccentrically in an attempt to confine any V1 activity to one quadrant of retinotopic cortex, thereby minimizing the occurrence of self-cancelling magnetic fields that may arise because of the cruciform architecture of primary visual areas. Figure 8 shows, for Observer A, SAM images of power decreases (purple-white colours) within the 10–20 Hz band for the passive perception of Glass patterns. The summed responses to radial, rotational and horizontal pattern types are shown because the neural responses to each pattern type were located in the same region. The top panels show the neural response to Glass patterns viewed centrally whereas the bottom panels show comparable peaks when patterns were viewed in the lower-left visual field. Note that both viewing conditions yielded loci of power decreases within the same visual area, close to V3a, and no significant activity was evident within area V1.

## Discussion

We used MEG to determine the neural signature associated with the perception of global structure from local image features. Our results provide evidence to suggest that the perception of global structure involves regions within or close to extrastriate visual area V3a (Figures 4–5, Table 1). This is based on data showing a reduction in low-frequency (10–20 Hz) oscillatory activity within that area, a neuroimaging attribute believed to indicate regions of heightened neural activity [e.g. 36], [ 41]–[44]. For example, using both language fluency and visual biological motion tasks, Singh et al. [36] showed that cortical areas with increased BOLD signal, as measured using fMRI, also had reduced beta power, as measured with MEG. More recently, Winterer et al. [43] confirmed that decreases in beta band power occurred at the same location as increases in BOLD signal, although they also report a complex set of increases and decreases in oscillatory power within different frequency bands. Similarly, Kinsey et al. [44] reported decreases in beta band power as the predominant MEG response to illusory visual contours. Such decreases in beta band activity, together with increases in power at higher frequencies, are predicted to occur within active neural areas, based on modeling the effects of factors such as changes in cell membrane properties and the coupling of neuronal assemblies [41].

Area V3a is intermediate between primary visual areas and higher-order visual areas within the inferotemporal cortex [7]. For several years area V3a has been considered a coherent motion processing area [2], [7], [10], [45], [46]. Dynamic Glass patterns contain no true coherent motion, and the flickering of the stimuli was unchanged during the active and baseline phases of the experiment (see Methods). However, it is possible that the sensitivity of this area to *perception* of motion underlies the activation we observed here as similar dynamic Glass patterns are known to induce a compelling illusion of motion [47], [48]. Indeed, whilst the concentric patterns appeared to swirl, and the radial to expand or contract, the horizontal patterns produced a weaker illusion of sideways motion (personal observations), and this may explain the trend for lesser cortical activity in response to horizontal patterns (discussed below). Alternatively, more recent neuroimaging studies have shown the involvement of V3a in form processing without motion [8], [9] and so the activations we observed may indeed reflect global form perception. It should also be noted that individual variations in the locations of visual areas make it difficult to distinguish spatially between them [40]. Due to this we must also remain open to the possibility that the active region was a neighbouring visual area such as V3. Consistent with this, an fMRI experiment using static Glass pattern stimuli reported activation of an area slightly inferior to V3a [1].

While the location of cortical activity was the same for radial, rotational and horizontal Glass patterns, we suggest the extent of activity was not. Our MEG data provides evidence that the perception of horizontal structure elicits less neural activity than the perception of either radial or rotational structure, at least with regard to oscillatory activity within the 10–20 Hz band (Figures 5 and 6). The differences in neural activation reported here are consistent with various reports of reduced perceptual sensitivity to horizontal patterns compared with rotational or radial patterns [15]–[19]. The reason for such differences has been debated for some time [e.g. 18], [ 21]. Dakin and Bex [21] attributed the observed differences to stimulus artifacts such as the shape of the stimulus aperture, noting that commonly used circular windows will bias detection for radial and rotational patterns. However, using the same experimental set-up as that employed here, where all stimuli were spatially confined using a square window, Anderson and Swettenham [14] demonstrated that the percentage of signal dot pairs required for the threshold detection of global structure is less for rotational and radial patterns than for horizontal patterns [xsee Figure 8 in [14]]. Together, these results indicate that different neural processes may underlie the perception of horizontal as opposed to radial or rotational structure. One possibility, proposed by Wilson and Wilkinson [18], is that we may be better at detecting radial and rotational structure because global summating mechanisms exist for their detection, whereas the detection of horizontal structure relies on local summating mechanisms. Another possibility is that only radial and rotational Glass patterns confer a well-defined centre and an impression of depth, visual attributes which may result in greater neural activity.

In our study, we did not observe any peaks of activity within primary visual cortex (V1) to the perception of global form in Glass patterns. To some extent, this was to be expected because area V1 is known to be sensitive to low-level image features, such as luminance and contrast, which we kept constant throughout the recordings (see Methods). Another possible reason for the lack of V1 activity relates to its anatomical structure, and the nature of magnetic fields. Despite inter-observer variability in cortical anatomy [49], V1 can generally be modeled as a cruciform structure because the calcarine and inter-hemispheric midline tend to form a cross at the occipital pole, with each quadrant of V1 responsive to a single quadrant of the visual field. In consequence, centrally-viewed stimuli may yield self-cancelling magnetic fields. To control for this possibility, we repeated the experiments using stimuli confined to a single quadrant of the field. Under such conditions, however, we still failed to identify V1 as an area of peak activity (Figure 8). Moreover, an absence of V1 activity to global form in Glass patterns was also noted using fMRI, where such anatomical considerations are not pertinent (unpublished observations).

Previous experimental evidence on the extent of V1 activity to global form perception is contradictory. An absence of V1 activity to global form was noted by both Braddick et al. [2] and Wilkinson et al. [11]. Other studies reported an increase in V1 activity to global form [50]–[52], while others still reported a decrease in activity [53], [54]. To some extent the conflicting findings on the involvement of V1 may be a reflection of the various tasks employed. For example, experiments requiring observers to attend to local features were associated with enhanced activity in V1 [50], [51], whereas passive viewing paradigms (such as that used here) were associated with either a decrease [53], [54] or unaltered activity [2], [11] within V1. Given our own results, we concur with Braddick et al. [2] that the perception of global structure from local features occurs beyond V1, and the evidence presented in this paper leads us to conclude that a candidate area for this process is V3a. This is consistent with recent evidence that the extraction of local orientation cues must precede the perception of global structure [55].
